# Changes in Liver Function Tests, Congestion, and Prognosis After Acute Heart Failure: The STRONG-HF Trial

**DOI:** 10.1016/j.jacadv.2025.101607

**Published:** 2025-02-21

**Authors:** Peder L. Myhre, Avishay Grupper, Alexandre Mebazaa, Beth Davison, Christopher Edwards, Koji Takagi, Marianna Adamo, Mattia Arrigo, Marianela Barros, Jan Biegus, Jelena Celutkiene, Kamilė Čerlinskaitė-Bajorė, Ovidiu Chioncel, Alain Cohen-Solal, Albertino Damasceno, Benjamin Deniau, Rafael Diaz, Gerasimos Filippatos, Etienne Gayat, Antoine Kimmoun, Jozine M. ter Maaten, Marco Metra, Maria Novosadova, Matteo Pagnesi, Peter S. Pang, Piotr Ponikowski, Hadiza Saidu, Karen Sliwa, Daniela Tomasoni, Adriaan Voors, Gad Cotter, Carolyn S.P. Lam

**Affiliations:** a K.G. Jebsen Center for Cardiac Biomarkers, University of Oslo, Oslo, Norway; b Division of Cardiology, Shamir Medical Center Beer Yakov, Be'er Ya'akov, Israel; c School of Medicine, Faculty of Medical and Health Sciences, Tel-Aviv University, Tel-Aviv, Israel; d Université Paris Cité, INSERM UMR-S 942 (MASCOT), Paris, France; e Department of Anesthesiology and Critical Care and Burn Unit, Saint-Louis and Lariboisière Hospitals, FHU PROMICE, DMU Parabol, APHP Nord, Paris, France; f Momentum Research, Durham, North Carolina, USA; g Heart Initiative, Durham, North Carolina, USA; h Cardiology, ASST Spedali Civili and Department of Medical and Surgical Specialties, Radiological Sciences, and Public Health, University of Brescia, Brescia, Italy; i Department of Internal Medicine, Zurich, Switzerland; j Institute of Heart Diseases, Wroclaw Medical University, Wroclaw, Poland; k Clinic of Cardiac and Vascular Diseases, Institute of Clinical Medicine, Faculty of Medicine, Vilnius University, Vilnius, Lithuania; l Emergency Institute for Cardiovascular Diseases "Prof C C Iliescu", University of Medicine "Carol Davila", Bucharest, Romania; m Université Paris Cité, INSERM UMR-S 942 (MASCOT), Paris, France; n APHP Nord, Department of Cardiology, Lariboisière University Hospital, Paris, France; o Faculty of Medicine, Eduardo Mondlane University, Maputo, Mozambique; p Estudios Clínicos Latinoamérica, Instituto Cardiovascular de Rosario, Rosario, Argentina; q National and Kapodistrian University of Athens, School of Medicine, Attikon University Hospital, Athens, Greece; r Université de Lorraine, Nancy, INSERM, Défaillance Circulatoire Aigue et Chronique, Service de Médecine Intensive et Réanimation Brabois, CHRU de Nancy, Vandœuvre-lès-Nancy, France; s University of Groningen, Department of Cardiology, University Medical Centre Groningen, Groningen, the Netherlands; t Department of Emergency Medicine, Indiana University School of Medicine, Indianapolis, Indiana, USA; u Murtala Muhammed Specialist Hospital, Bayero University Kano, Kano, Nigeria; v Division of Cardiology, Department of Medicine, Groote Schuur Hospital, University of Cape Town, Cape Town, South Africa; w National Heart Centre Singapore and Duke-National University of Singapore, Singapore, Singapore; x Baim Institute for Clinical Research, Boston, Massachusetts, USA; y University Medical Centre Groningen, Groeningen, the Netherlands

**Keywords:** acute heart failure, ALT, high intensity care, liver function tests

## Abstract

**Background:**

Elevated alanine aminotransferase (ALT), aspartate aminotransferase (AST), and total bilirubin (tBil) may reflect congestion and liver dysfunction in acute heart failure (AHF), while lower ALT also associates with sarcopenia.

**Objectives:**

The purpose of this study was to assess ALT, AST, and tBil levels in AHF patients during high-intensity care (HIC) vs usual care (UC) follow-up.

**Methods:**

ALT, AST, and tBil were measured 1 to 2 days predischarge in 1,062 AHF patients, and again after 90 days of either HIC or UC according to the STRONG-HF (Safety, Tolerability and efficacy of Rapid Optimization, helped by NT-proBNP testinG, of Heart Failure therapies) protocol. The primary endpoint was 180-day all-cause death or HF hospitalization.

**Results:**

Median (Q1-Q3) baseline ALT, AST, and tBil were 21 (15-32) U/L, 23 (17-32) U/L, and 14(10-21) umol/L, respectively. Patients with lower ALT had lower body mass index. Patients with lower ALT, but not tBil or AST, were more likely to have edema, elevated jugular venous pressure, and orthopnea, and use more diuretics prerandomization. A nonsignificant inverse association between ALT and the primary outcome (HR: 0.82 [95% CI: 0.66-1.01] per log-unit, *P* = 0.06) was observed. Greater reductions of ALT, AST, and tBil to 90 days were associated with greater improvement of edema, rales, NYHA functional class, and N-terminal pro-B-type natriuretic peptide. After 90 days, the HIC group had a greater reduction in AST and tBil than the UC group, while nonsignificant for ALT. The treatment effect of HIC over UC on the primary outcome was consistent across the baseline ALT, AST, and tBil range (all *P* interaction >0.10), but patients with lower ALT experienced greater health status improvement from HIC.

**Conclusions:**

Lower ALT was associated with lower body mass index and more congestion in AHF, supporting previous studies suggesting ALT as a sarcopenia marker. The beneficial effect of HIC on health status was greater in low baseline ALT patients. (Safety, Tolerability and Efficacy of Rapid Optimization, Helped by NT-proBNP testinG, of Heart Failure Therapies [STRONG-HF]; NCT03412201)

Acute heart failure (AHF) represents a clinical syndrome that engenders a cascade of systemic effects due to impaired cardiac function and the ensuing elevated left ventricular filling pressure. Central to these systemic manifestations is the impact of right-sided congestion of visceral organs, notably affecting the liver, spleen, kidney, and gastrointestinal tract.^1^, ^2^, ^3^ The liver, in the setting of AHF, often exhibits deranged function as a consequence of passive congestion and hypoperfusion, which, in turn, can be detected and monitored through liver function tests (LFTs).^4^, ^5^, ^6^ These tests, which include markers such as alanine aminotransferase (ALT), aspartate aminotransferase (AST), and total bilirubin (tBil), among others, serve as indirect indicators of the hemodynamic burden on the liver as well as the AHF's severity and progression.^7^, ^8^, ^9^

Cachexia and sarcopenia are systemic conditions characterized by the loss of muscle mass with or without loss of fat mass. This is a feared complication in chronic diseases such as cancer and end-stage liver disease but has also been increasingly recognized in patients with HF as it severely affects patient quality of life, functional independence, and overall prognosis.^10^ Recent observations suggest a potential link between low levels of ALT and the presence of cachexia/sarcopenia,^11^ adding a layer of complexity to their role in evaluating patients with AHF.^12^ We therefore aimed to assess the association between LFTs and the clinical presentation, including congestion status, and outcomes in patients hospitalized for AHF in the STRONG-HF (Safety, Tolerability and efficacy of Rapid Optimization, helped by NT-proBNP testinG, of Heart Failure therapies) trial.^13^ We also aimed to assess trajectories of LFTs during rapid up-titration of HF guideline-directed medical therapy (GDMT) as compared to usual care (UC).

## Methods

This study is a post hoc analysis of the STRONG-HF trial, of which the design and main results have previously been reported.^13^

In short, this was a multicenter, open-label, randomized trial of 1,078 patients admitted to the hospital for AHF who were not treated with optimal doses of GDMT. Within 2 days before anticipated discharge, patients were randomized 1:1 to either UC or a high-intensity care (HIC) strategy with early up-titration of beta-blockers, renin-angiotensin system renin-angiotensin system inhibitors (angiotensin-converting enzyme inhibitors or angiotensin receptor blockers in patients intolerant to angiotensin-converting enzyme inhibitor, or angiotensin receptor-neprilysin inhibitor), and mineralocorticoid receptor antagonists. Eligible patients had to be between 18 and 85 years old, hemodynamically stable, and with elevated N-terminal pro-B-type natriuretic peptide (NT-proBNP) levels. Patients with a primary liver disease considered to be life-threatening were excluded from STRONG-HF, and for the purpose of this substudy, six enrolled patients with a medical history of severe liver disease were also excluded from these analyses. There were no eligibility restrictions in left ventricular ejection fraction (LVEF). Patients in the HIC group had follow-up visits at 1, 2, 3, and 6 weeks and at day 90 after randomization, including physical examination to assess congestion and laboratory assessments. Doses of beta-blockers, renin-angiotensin system inhibitors, and mineralocorticoid receptor antagonists were up-titrated in the HIC group to half optimal doses at randomization and to full optimal doses at week 2. Patients in the UC group were followed up according to local practice until a study visit performed at day 90 after randomization. All randomized patients were contacted at day 180 to assess the occurrence of rehospitalizations and death. The trial's primary endpoint was 180-day all-cause death or readmission for HF. The secondary endpoint of this study was change in health-related quality of life (HRQoL) assessed by the EuroQol 5-dimension (EQ-5D) visual analog scale at baseline and again after 90 days. The study was approved by appropriate competent authorities and all sites obtained approval from the ethics committees. All patients provided written informed consent. The study is registered at ClinicalTrials.gov, NCT03412201.

Concentrations of ALT, AST, and tBil were analyzed locally from blood samples collected at randomization and at the day 90 visit. In the HIC group, measurements of ALT, AST, and tBil were also performed at the 1, 2, 3, and 6 week visits.

### Statistical analysis

Categorical variables are presented as number and percentage, and continuous variables as mean ± SD or median (25th, 75th percentiles). Comparisons of baseline characteristics for trend across quartiles of ALT, AST, and tBil were performed by Jonckheere–Terpstra for continuous variables, Cochran–Armitage for binary variables, and Cochran–Mantel–Haenszel for categorical variables. When assessing associations between changes in LFT levels from baseline to 90 days and changes in congestion markers, patients were grouped in categories of 5 U/L or greater increase or decrease for ALT and AST, and categories of 3 U/L or greater increase or decrease for tBil. Comparisons between treatment groups regarding changes in vital signs and laboratory measures were assessed by analysis of covariance models adjusted for baseline value, and randomization stratification factors (geographic region and LVEF ≤/> 40%). When considered as continuous variables, AST, ALT, and tBil values, which were right-skewed, were log-transformed for analysis.

Analyses of endpoints through 180 days were restricted to patients enrolled at sites where the ethics committee approved the amended protocol allowing follow-up of patients through day 180, and in the cohort of patients enrolled before the primary endpoint was changed from 90 to 180 days the results were down-weighted proportional to half its sample size. Cox proportional hazards regression analysis was performed to evaluate the impact of log-transformed levels of ALT, AST, and tBil measured at baseline on the primary outcome, presented as HR and 95% CI. The association between levels of each biomarker and the outcome was tested for nonlinearity, which was not statistically significant for any. The treatment effect of HIC vs UC on the primary endpoint was modeled as a function of continuous baseline levels of ALT, AST, and tBil, as a restricted cubic spline using three knots. The potential modification of the treatment effect on EQ-5D visual analog scale by baseline levels of ALT, AST, and tBil was assessed using analysis of covariance with adjustment for baseline EQ-VAS and randomization stratification factors (LVEF ≤40/>40% and geographic region). Analyses were restricted to countries where a linguistically validated translation was used (ie, excludes subjects from Mozambique). Two-sided *P* values <0.05 were considered to be statistically significant. All analyses were performed using SAS, version 9.4 (SAS Institute).

## Results

### Baseline characteristics

In this substudy of STRONG-HF, the mean age was 63 ± 14 years, 39% were female, 77% White or Caucasian, 68% had LVEF ≤40%, and the median (Q1-Q3) NT-proBNP concentration was 2,868 (1,918-4,973) pg/mL at randomization (baseline). ALT, AST, and tBil measurements were available in 1,062, 1,057, and 1,005 participants (98.5%, 98.1%, and 93.2% of all STRONG-HF participants, respectively), and the median (Q1-Q3) concentrations were 21 (15-32) U/L, 23 (17-32) U/L, and 14 (10-21) umol/L, respectively, at baseline. The correlation coefficients between levels of these biomarkers at baseline were ALT-AST 0.68, ALT-tBil 0.20, and AST-tBil 0.29, all *P* < 0.001.

### Associations of ALT, AST and tBil with baseline clinical characteristics

**Figure 1:**
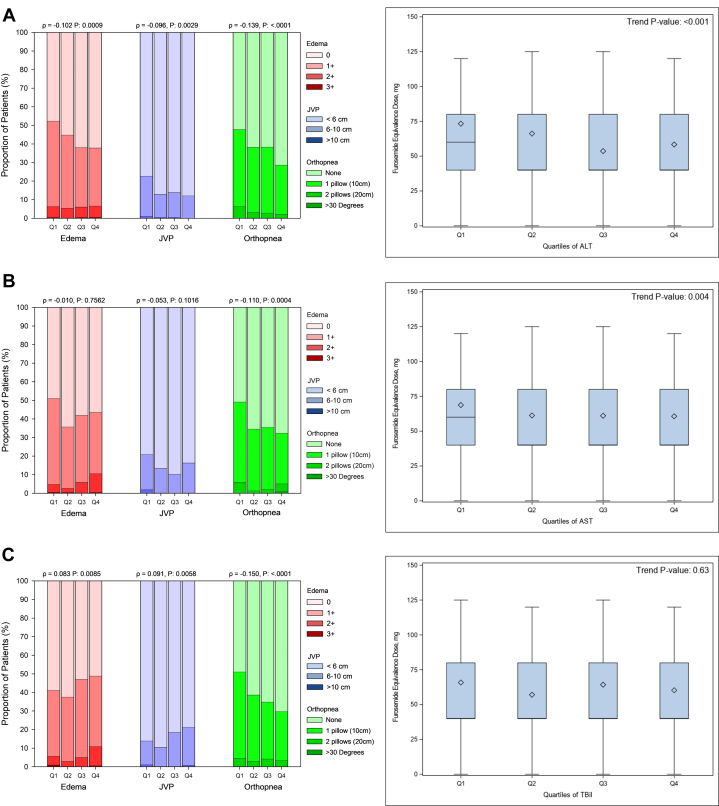
**Association Between Baseline Liver Function Tests and Markers of Congestion** Association between quartiles of baseline liver function tests and clinical markers of congestion and furosemide equivalence doses. (A) ALT, (B) AST, (C) Total Bilirubin. ALT = alanine aminotransferase; AST = aspartate aminotransferase; tBil = total bilirubin.

Lower concentrations of ALT, AST, and tBil at baseline were associated with female sex, Black race, lower body mass index (BMI), higher LVEF, lower hemoglobin, lower creatinine, and less frequent atrial fibrillation/flutter, while there were no significant associations with age. (Table 1, Supplemental Tables 1 and 2). Patients with low ALT were more likely to have (presented Q1 vs Q4 and *P* value for correlation) peripheral edema (52.5% vs 38.3%, *P* < 0.001), elevated jugular venous pressure (JVP) (22.9% vs 12.5%, *P* = 0.002), orthopnea (47.9% vs 28.8%, *P* < 0.001), and use higher loop diuretic doses (73 ± 59 mg vs 58 ± 39 mg, *P* < 0.001) (Figure 1). In contrast, patients with low tBil had less peripheral edema and lower JVP. AST was inversely correlated with orthopnea but was not significantly correlated with other signs of congestion. Baseline patient-reported health status tended to be better in patients with higher baseline LFT levels. Higher EQ-VAS was modestly but statistically significantly positively correlated with higher baseline ALT (r = 0.13, *P* < 0.001), AST (r = 0.17, *P* < 0.001), and tBil (r = 0.09, *P* = 0.004).

**Table 1:** Baseline Characteristics at Time of Randomization by Quartiles of ALT

	ALT <15.1 U/L (n = 272)	ALT 15.1-21.0 U/L (n = 261)	ALT 21.1-31.9 U/L (n = 261)	ALT >31.9 U/L (n = 268)	Trend *P* Value^a^
Age, y	62.7 ± 15.07	62.5 ± 14.08	63.9 ± 11.85	62.1 ± 13.11	0.29
Female	134 (49.3%)	107 (41.0%)	83 (31.8%)	85 (31.7%)	<0.001
Black race	84 (31.0%)	68 (26.1%)	41 (15.7%)	36 (13.4%)	<0.001
Stroke or transient ischemic attack	24 (8.9%)	29 (11.1%)	23 (8.8%)	22 (8.2%)	0.59
Diabetes	82 (30.1%)	80 (30.7%)	79 (30.4%)	68 (25.6%)	0.26
Acute coronary syndrome	76 (27.9%)	76 (29.1%)	76 (29.1%)	82 (30.6%)	0.52
History of heart failure	240 (88.2%)	224 (85.8%)	227 (87.0%)	215 (80.2%)	0.02
Left ventricular ejection fraction, %	38.7 (13.29)	35.9 (11.89)	36.2 (11.99)	34.2 (12.35)	<0.001
Hospitalized for heart failure past year	73 (26.8%)	67 (25.7%)	63 (24.1%)	67 (25.0%)	0.55
Atrial fibrillation or flutter	111 (40.8%)	110 (42.1%)	130 (49.8%)	138 (51.5%)	0.004
Systolic blood pressure, mm Hg	122.7 ± 13.33	123.7 ± 12.53	123.8 ± 12.85	121.5 ± 12.85	0.30
Pulse, beats/min	78.6 ± 11.30	78.9 ± 11.70	77.3 ± 10.79	79.6 ± 12.98	0.923
BMI (kg/m^2^)	27.5 ± 6.27	27.9 ± 6.13	28.9 ± 6.45	28.9 ± 5.93	<0.001
Hemoglobin, g/L	129.0 ± 17.87	135.2 ± 19.23	138.5 ± 19.49	143.1 ± 20.68	<0.001
Creatinine, umol/L	103.3 ± 32.12	106.2 ± 27.60	107.7 ± 30.55	108.0 ± 25.10	0.002
Potassium, mmol/L	4.2 ± 0.49	4.3 ± 0.44	4.3 ± 0.44	4.3 ± 0.41	0.002
Sodium, mmol/L	139.4 ± 4.00	140.5 ± 3.94	140.3 ± 4.19	140.7 ± 4.40	<0.001
NT-proBNP, ng/L	3,221 (2,995-3,465)	2,959 (2,749-3,186)	3,403 (3,148-3,678)	3,326 (3,070-3,604)	0.26
ACE inhibitors/ARBs/ARN inhibitors	185 (68.3%)	173 (66.3%)	169 (65.0%)	155 (58.1%)	0.015
ß-blockers	78 (28.8%)	90 (34.5%)	93 (35.8%)	117 (43.8%)	<0.001
Mineralocorticoid receptor antagonists	255 (94.1%)	249 (95.4%)	243 (93.5%)	260 (97.4%)	0.18
Furosemide equivalence dose, mg	73.3 ± 59.13	66.2 ± 45.97	53.6 ± 35.75	58.4 ± 38.58	<0.001

ACE = angiotensin-converting enzyme; ALT = alanine aminotransferase; ARBs = angiotensin receptor blockers; ARN = angiotensin receptor-neprilysin; BMI = body mass index; NT-proBNP = N-terminal pro-B-type natriuretic peptide.

Values are mean ± SD, n (%), or geometric mean (95% CI).

a Jonckheere's trend test for continuous variables, Cochron-Armitage trend test for binary variables, Cochran-Mantel-Haenszel general association for categorical variables, and Cochran-Mantel-Haenszel nonzero correlation for ordinal variables.

### Baseline ALT, AST and tBil in association with outcomes and changes in health status

There was a nonsignificant trend toward an increased risk of death or HF hospitalization for patients with lower ALT levels: HR: 0.82 (95% CI: 0.66-1.01) per log increase, *P* = 0.06 (Supplemental Figure 1; Central Illustration). This association was not changed after adjusting for age, sex, race, BMI, LVEF, NT-proBNP, and treatment arm (HR: 0.81 [95% CI: 0.65-1.01] per log increase, *P* = 0.06). Baseline levels of AST and tBil were not significantly associated with prognosis; HR: 0.87 (95% CI: 0.70-1.09) per log increase, *P* = 0.22 and HR: 1.05 (95% CI: 0.85-1.29) per log increase, *P* = 0.67, respectively. This persisted after adjustment: HR: 0.82 (95% CI: 0.66-1.02), *P* = 0.08 for AST and HR: 1.02 (95% CI: 0.81-1.27), *P* = 0.88 for tBil.

**Central Illustration:**
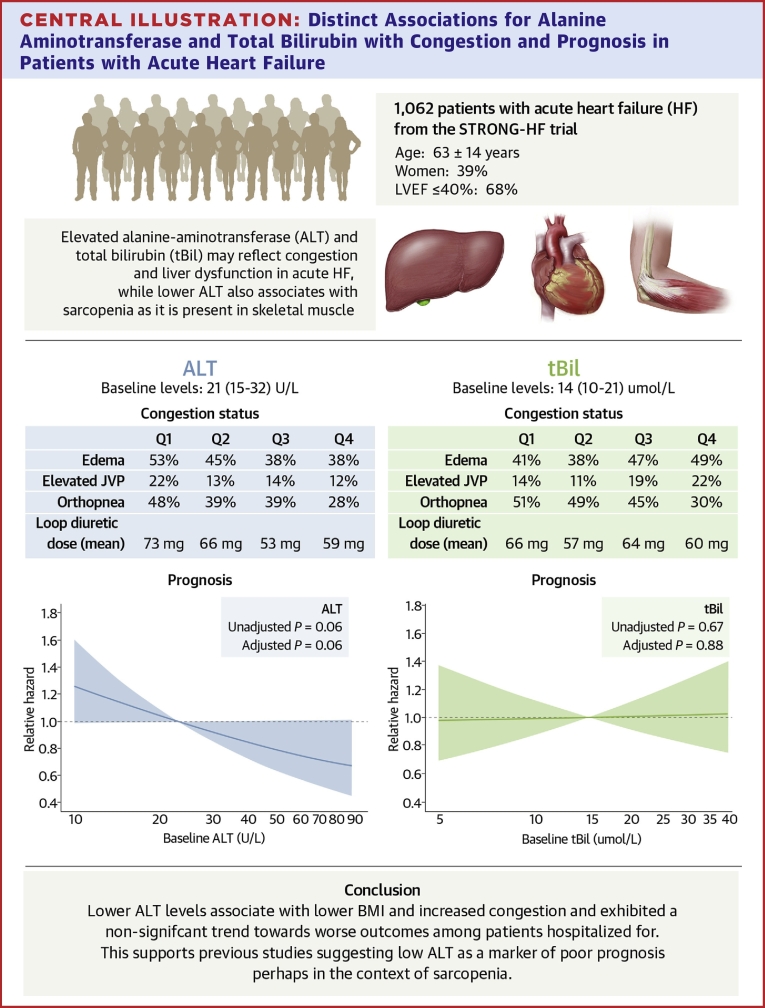
Distinct Associations for Alanine Aminotransferase and Total Bilirubin with Congestion and Prognosis in Patients with Acute Heart Failure

Among all STRONG-HF study participants, baseline concentrations of ALT, AST, and tBil were not statistically significantly associated with the change in EQ-VAS from baseline to day 90 (*P* = 0.13, 0.55, and 0.39, respectively) (Supplemental Figure 2).

### Changes in ALT, AST and tBil in association with changes in markers of congestion

After 90 days, measurements of ALT, AST, and tBil were available in 901, 907, and 888 patients and the median (Q1-Q3) concentrations were 20 (14-28) U/L, 21 (17-28) U/L, and 12 (9-17) umol/L, respectively. In general, greater reductions in AST, ALT, and tBil from baseline to day 90 were associated with greater reductions in peripheral edema, JVP, rales, NYHA class, and NT-proBNP (Supplemental Tables 3A to 3C). However, the correlations were modest with Rho ranging from 0.02 to 0.15.

### Rapid up-titration of GDMT by baseline levels of ALT, AST and tBil

**Figure 2:**
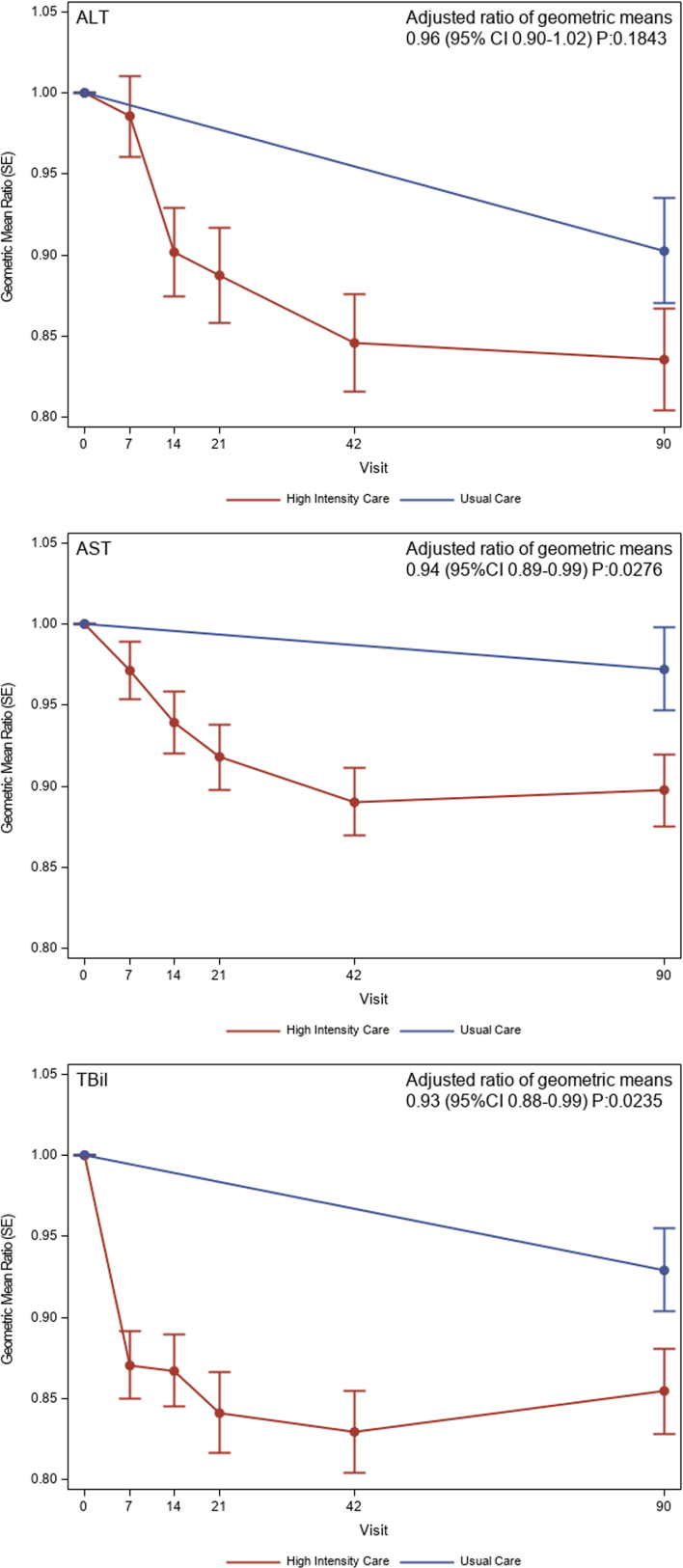
Changes in Liver Function Tests in the High-Intensity Care and Usual Care Groups Profile plot of trajectory of changes in liver function tests from baseline to follow-up in high-intensity care and usual care. Abbreviations as in Figure 1.

**Figure 3:**
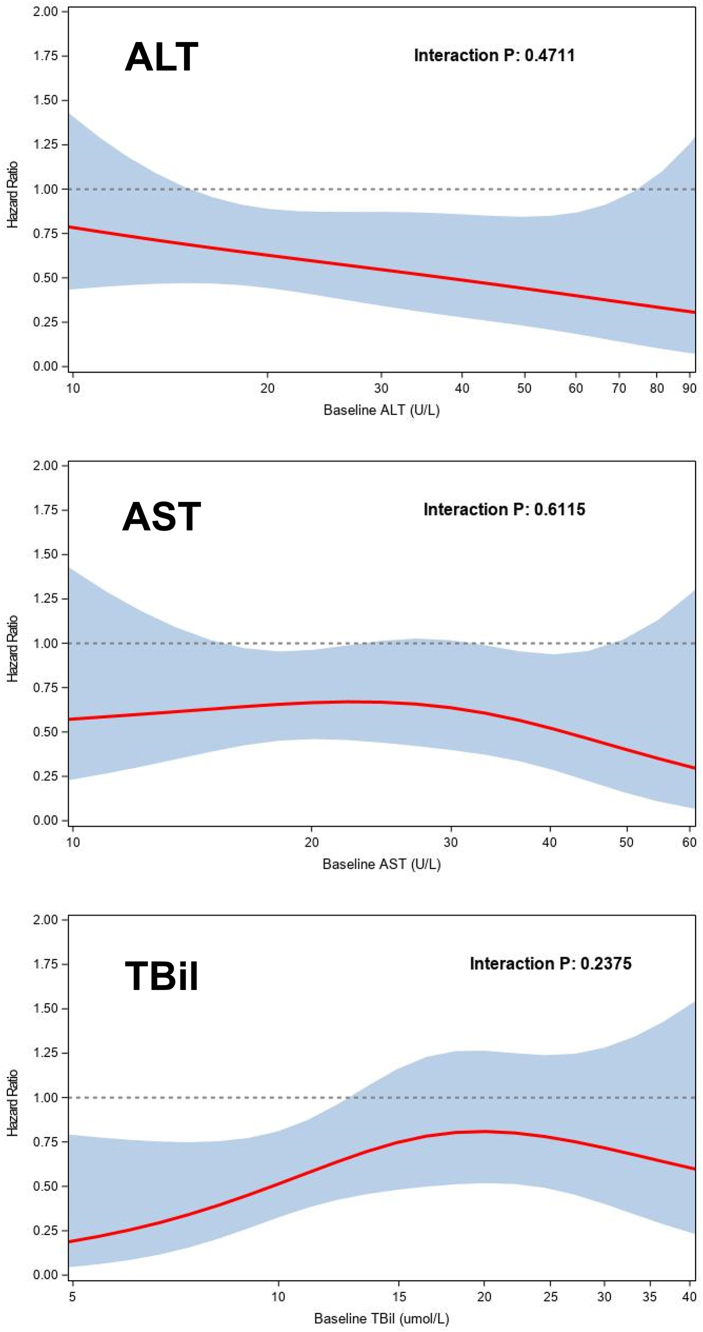
Treatment Effect of High-Intensity Care vs Usual Care by Baseline Liver Function Test Levels Treatment effect of high-intensity care vs usual care on the primary endpoint of death or heart failure readmission through day 180 according to baseline liver function tests. Abbreviations as in Figure 1.

Patients randomized to HIC experienced a greater reduction in AST and tBil (ratio of geometric means of day 90 relative to baseline: 0.94 [95% CI: 0.89-0.99] *P* = 0.028 and 0.93 [95% CI: 0.88-0.99] *P* = 0.024, respectively) compared to UC, while there was no significant difference in ALT (0.96 [95% CI: 0.90-1.02] *P* = 0.18) (Figure 2). The benefit of HIC over UC in reducing death or HF hospitalization did not vary significantly across the range of ALT (*P* for interaction = 0.48), AST (*P* = 0.63), and tBil (*P* = 0.16) (Figure 3). Improvements in HRQoL to 90 days by the HIC over UC were more pronounced in patients with lower ALT (mean change in EQ-5D points 6.5 in Q1, 5.4 in Q2, 2.8 in Q3 and -0.4 in Q4, *P* for interaction = 0.032), while no significant associations were found between HRQoL and AST (*P* = 0.07) and tBil (*P* = 0.43). Results were similar when the LFTs were examined as continuous variables (interaction *P* = 0.030, 0.056, and 0.14, respectively; Supplemental Figure 3). The occurrence of adverse events from HIC vs UC did not differ by baseline ALT, AST, or tBil (*P* for interaction = 0.40, 0.67, and 0.059, respectively), with equivalent results for serious adverse events (*P* for interaction = 0.42, 0.70, and 0.78, respectively). There was no clear pattern of impaired up-titration (reaching target doses) by baseline levels of ALT, AST, and tBil.

## Discussion

This study of patients with AHF has 6 findings. 1) Lower ALT levels at hospital discharge were associated with lower BMI and more congestion, a pattern not observed for tBil. 2) There was a nonsignificant trend indicating worse outcomes in patients with lower ALT levels at hospital discharge, a trend not observed for tBil levels. 3) At 90 days, greater reductions of ALT, AST, and tBil were associated with greater improvement of edema, rales, NYHA class, and NT-proBNP. 4) Rapid up-titration of GDMT after hospital discharge resulted in statistically significantly greater reductions in AST and tBil compared to UC but not in ALT. 5) The prognostic benefit of HIC over UC was present irrespective of baseline ALT, AST, and tBil levels. 6) HRQoL improvements at 90 days with HIC over UC were more pronounced in patients with lower ALT. The clinical implication of these findings is that elevated ALT should not be automatically regarded as a marker of liver congestion and worse outcome in AHF, as is often assumed. Considering the complex interplay between reduced ALT expression due to sarcopenia and increased ALT expression from liver failure, it is crucial to interpret this biomarker with caution. Understanding these dynamics will aid clinicians in making more informed decisions regarding patient prognosis and treatment strategies in AHF.

Our finding suggested an inverse association between ALT and outcomes, which contrasts with some previous studies in AHF.^2^^,^^5^^,^^14^ In several observational studies, elevated liver tests at admission have tended to be associated with a higher in-hospital mortality, but these cohorts included very diverse patients with hemodynamic abnormalities, high prevalence of cardiogenic shock, and high proportion of patients treated with intravenous inotropes.^3^^,^^5^^,^^15^^,^^16^ Furthermore, ALT values have predominantly been considered at hospital admission and not predischarge as in STRONG-HF. Although this may explain part of the diverging results, data from the EVEREST (Efficacy of Vasopressin Antagonism in Heart Failure: Outcome Study With Tolvaptan) study suggest that liver enzymes remained practically unchanged from admission for AHF to discharge.^17^ In the SURVIVE (Utility of Serelaxin in Treating Acute Heart Failure) trial, elevated admission ALT or AST (defined by an upper normal limit of 47 U/L for ALT and 37 U/L for AST) was associated with an increased risk of mortality.^7^ This study enrolled patients with severe and hemodynamically unstable AHF requiring inotropic support (27% 180-day mortality), in contrast to STRONG-HF, which enrolled patients who were stabilized and ready for discharge (7% 180-day mortality). In SURVIVE, ALT and AST were above upper normal limit in 25% for ALT and 33% for AST, while only 11% and 16% of patients exceeded these thresholds in STRONG-HF. As, LFT abnormalities may stem from different central hemodynamic abnormalities, the laboratory profiles may differ in different HF phenotypes or stages. Patients with low cardiac output are more likely to develop a rise in aminotransferases, while those with predominant congestion should present more profound abnormalities in bilirubin or gamma-glutamyl transpeptidase.^4^^,^^9^ This may explain the differences between the biomarker profile in each of the AHF studies. This is supported by the fact that elevated ALT/AST in SURVIVE was associated with *less* peripheral edema and ascites, and with higher pulse, lower blood pressure, and the presence of cold extremities. Also notable is that acute myocardial infarction was the reason for ADHF in 31% of patients with elevated ALT/AST, compared to 9% in those with normal levels, most likely reflecting that a proportion of the circulating concentrations originate from myocardial injury. Of note, decreasing ALT and AST from baseline to day 5 in SURVIVE was not associated with improved survival, in fact, numerically more patients with decreasing ALT died, as compared to increasing ALT.

Studies of less severe AHF have demonstrated inconsistent results with respect to LFTs.^18^ In a retrospective analysis of >11,000 patients with AHF at a large tertiary care hospital in Israel, patients with low ALT (<10 U/L) had worse survival.^19^ Patients with low ALT were older and more frequently had a history of stroke, dementia, and malignancy, suggesting increased frailty. Of note, this study excluded patients with ALT >40 U/L or liver cirrhosis (n = 2,359). In the ASCEND-HF (Acute Study of Clinical Effectiveness of Nesiritide in Decompensated Heart Failure) trial, higher tBil was associated with an increased risk of mortality, while ALT and AST were not.^8^ ASCEND-HF enrolled lower risk AHF patients than SURVIVE (4% 30-day mortality) who were quite stable as patients with hypotension were excluded. In line with our findings, higher ALT was associated with less peripheral edema in ASCEND-HF. Of note, the liver enzymes assessments in ASCEND were done close to admission and not predischarge.^8^

A key novel aspect of our study involves identifying AHF patients who are more likely to benefit from HIC. Although we did not find that ALT (or AST and tBil) modified the effect from HIC vs UC on cardiovascular events, we did observe a significant effect modification by ALT on HRQoL. Specifically, patients with lower ALT at baseline experience greater benefits. A similar, though not statistically significant, trend was seen for AST and tBil. This again suggests that lower ALT reflects sicker patients who stand to gain more from rapid up-titration and close monitoring postdischarge. This provocative finding challenges the common belief that patients with high ALT levels are more congested and decompensated and should therefore be prioritized for intense monitoring and treatment. It is, however, important to acknowledge that the temporal *change* in LFTs during follow-up reflects congestion in the expected direction. Patients with a reduction in ALT, AST, and tBil experienced more decongestion than patients with stable or increasing levels. Moreover, patients in the HIC group had greater reductions in LFTs compared to the UC group, in line with a superior effect of HIC on congestion and NT-proBNP reductions.^13^^,^^20^^,^^21^ This association was more pronounced for AST and tBil than for ALT, probably reflecting the association between each biomarker and congestion.

The relationship between ALT and cachexia is complex and not fully understood. Traditionally, ALT is considered a liver enzyme that is released into the bloodstream as an indicator of liver cell damage. However, its levels and behavior might differ significantly in the context of systemic illnesses like cachexia. In cachexia, which is characterized by severe muscle wasting and weight loss, levels of ALT are observed to be low. In the NHANES (National Health and Nutrition Examination Survey) III study (mean age 45 years), lower ALT was associated with a lower appendicular lean mass measured by dual-energy x-ray absorptiometry body composition measures, a lower physical activity level, and an increased risk of death.^11^ Potential explanations for the link between cachexia and ALT include reduced muscle mass (sarcopenia), as ALT also is present in skeletal muscles, and a decrease in muscle mass due to cachexia might therefore lead to a reduction in the total amount of ALT released into the bloodstream.^22^ However, the association may also be explained by altered metabolism related to malnutrition and cachexia. The biochemical activity of ALT is to catalyze pyruvate to alanine in the skeletal muscle and alanine to pyruvate in the liver. As such, ALT could transform potential energy within muscle cells whenever pyruvate is not fully assimilated into the Krebs cycle by exporting alanine to the liver, where it is once again transformed by ALT to pyruvate and exploited as available energy. A poor metabolic status of the cell secondary to cachexia, including shifts in amino acid metabolism, could therefore impact the production and clearance rates of enzymes such as ALT.

### Study Limitations

The current study has limitations. First, we excluded six patients with severe liver disease in an attempt to get a purer analysis of LFTs in AHF. Second, there were a small proportion of patients with missing measurements of ALT, AST, and tBil at baseline and follow-up. Although a small proportion of missingness was due to death, the majority are assumed to be missing at random. Third, assessments of congestion may lack precision as they were made by each local investigator. Fourth, the study was unblinded, which might have affected the perceptions of the study team. A final limitation of this study is the reliance on BMI as an indicator of sarcopenia or cachexia, as it may not accurately reflect muscle mass or fat distribution. Further research using more precise body composition measurements is necessary to clarify the relationship between ALT levels and indicators of cachexia or sarcopenia.

## Conclusions

Lower ALT levels were associated with lower BMI and increased congestion and exhibited a nonsignificant trend toward worse outcomes among patients hospitalized for AHF in the STRONG-HF trial. This supports previous studies suggesting low ALT as a marker of poor prognosis perhaps in the context of sarcopenia. The beneficial effect of HIC on clinical outcomes was consistent irrespective of ALT, AST, and tBil at discharge, but patients with lower levels experienced greater improvements in health status from the intervention.

## Funding support and author disclosures

The STRONG-HF trial was funded by 10.13039/100016545Roche Diagnostics. Dr Myhre has received research grants from 10.13039/100004325AstraZeneca and speaker and/or consulting fees from Amarin, AmGen, AstraZeneca, Bayer, Boehringer Ingelheim, Bristol Myers Squibb, Novartis, Novo Nordisk, Orion Pharma, Pharmacosmos, Roche Diagnostics, Vifor, and Us2.ai. Dr Mebazaa has received grants from 10.13039/100016545Roche Diagnostics, 10.13039/100001316Abbott Laboratories, 4TEEN4, and 10.13039/100020390Windtree Therapeutics; honoraria for lectures from 10.13039/100016545Roche Diagnostics, 10.13039/100004326Bayer, and 10.13039/100007054MSD; is a consultant for Corteria Pharmaceuticals, S-form Pharma, FIRE-1, Implicity, 4TEEN4, and Adrenomed; and is a coinventor of a patent on combination therapy for patients having acute or persistent dyspnea. Drs Beth Davison, Edwards, Takagi, Barros, Novosadova, and Cotter are employees of Momentum Research, which has received grants for research from 10.13039/100001316Abbott Laboratories, 10.13039/100002429Amgen, Celyad, 10.13039/100017623Cirius Therapeutics, Corteria Pharmaceuticals, Heart Initiative, 10.13039/100004339Sanofi, 10.13039/100020390Windtree Therapeutics, and XyloCor Therapeutics. Drs BD and Cotter are directors of Heart Initiative, a nonprofit organization. Dr Adamo has received speaker fees from Abbott Vascular and Medtronic. Dr Celutkiene has received personal fees from Novartis, AstraZeneca, Boehringer Ingelheim, Roche Diagnostics, and Pfizer. Dr Chioncel received grants from Servier. Dr Cohen-Solal has received honoraria for lectures or consultancy from AstraZeneca, Novartis, Vifor, Bayer, Merck, Sanofi, Abbott, and Boehringer Ingelheim. Dr Damasceno works for the Faculty of Medicine, Eduardo Mondlane University (Maputo, Mozambique), which received research grants from the Heart Initiative for their participation in this study. Dr Diaz has received supporting fees for coordination of STRONG-HF trial activities. Dr Filippatos has received lecture fees or was a committee member for trials and registries sponsored by Bayer, Vifor, Boehringer Ingelheim, Medtronic, Servier, and Amgen. Dr ter Maaten reports speaker and/or consultancy fees to institution from Novartis, Boehringer Ingelheim, Moderna, Roche, and Novo Nordisk, and receiving grants from Netherlands Heart Foundation, and Netherlands Organization for Scientific Research (NOW) outside the submitted work. Dr Metra has received personal fees from Amgen, Livanova, and Vifor Pharma as a member of executive committees of sponsored clinical trials and from AstraZeneca, Bayer, Boehringer Ingelheim, Edwards Lifesciences, and Roche Diagnostics for participation to advisory boards or for speaking at sponsored meetings. Dr Pagnesi has received personal fees from Abbott Laboratories, AstraZeneca, Boehringer Ingelheim, and Vifor Pharma. Dr Pang has received grants or research contracts from American Heart Association, Roche, Siemens, Ortho Diagnostics, Abbott, Beckman Coulter, and Siemens; consulting fees from Roche; honoraria from WebMD; and he has financial interest in The Heart Course. Dr Ponikowski reports grants and personal fees from Amgen, grants and personal fees from Servier, Boehringer Ingelheim, Vifor Pharma, Novartis, Bayer, Cibiem, AstraZeneca, BMS, Renal Guard Solutions, Impulse Dynamics, and Abbott Vascular, and personal fees from Berlin Chemie outside of the submitted work. Dr Sliwa has received grants from Medtronic, Servier, and Amylam and honoraria from MSD, Novartis, and Sanofi. Dr Tomasoni has received speaker fees from Boehringer Ingelheim, Alnylam Pharmaceuticals, and from Pfizer. Dr Voors has received consultancy fees or research support from AstraZeneca, Bayer, BMS, Boehringer Ingelheim, Cytokinetics, Myocardia, Merck, Novartis, Novo Nordisk, and Roche Diagnostics. Dr Lam is supported by a Clinician Scientist Award from the National Medical Research Council of Singapore; has received research support from 10.13039/501100004191Novo Nordisk and 10.13039/100016545Roche Diagnostics; has served as consultant or on the Advisory Board/Steering Committee/Executive Committee for Alleviant Medical, Allysta Pharma, AnaCardio AB, Applied Therapeutics, AstraZeneca, Bayer, Boehringer Ingelheim, Boston Scientific, Bristol Myers Squibb, CardioRenal, CPC Clinical Research, Eli Lilly, Impulse Dynamics, Intellia Therapeutics, Ionis Pharmaceutical, Janssen Research & Development LLC, Medscape/WebMD Global LLC, Merck, Novartis, Novo Nordisk, Prosciento Inc, Quidel Corporation, Radcliffe Group Ltd, Recardio Inc, ReCor Medical, Roche Diagnostics, Sanofi, Siemens Healthcare Diagnostics, and Us2.ai; and serves as cofounder and nonexecutive director of Us2.ai. All other authors have reported that they have no relationships relevant to the contents of this paper to disclose.
