# Supplementary material for: Changes in Liver Function Tests, Congestion, and Prognosis After Acute Heart Failure: The STRONG-HF Trial
Source: JACC Adv. 2025 Feb 21;4(3):101607. doi: 10.1016/j.jacadv.2025.101607 (PMC11891714; doi:10.1016/j.jacadv.2025.101607)
Supplement: Supplemental material [file mmc1.docx]

**Supplemental Table 1. Baseline characteristics at time of randomization by quartiles of AST**

| **Parameter** | **AST < 17.4 U/L (N=264)** | **AST 17.4-22.8 U/L (N=263)** | **AST 22.9-31.9 U/L (N=264)** | **AST > 31.9 U/L (N=266)** | **Trend**  **P-value#** |  |
| --- | --- | --- | --- | --- | --- | --- |
|  |  |  |  |  |  |  |
| Age, years | 60.1 (15.64) | 63.6 (13.00) | 65.2 (12.28) | 62.4 (12.83) | 0.17 |  |
| Female sex | 125 (47.3%) | 108 (41.1%) | 97 (36.7%) | 79 (29.7%) | <0.001 |  |
| Black race | 96 (36.5%) | 49 (18.6%) | 38 (14.4%) | 45 (16.9%) | <0.001 |  |
| Stroke or transient ischemic attack | 20 (7.6%) | 33 (12.6%) | 25 (9.5%) | 19 (7.1%) | 0.57 |  |
| Diabetes | 72 (27.3%) | 90 (34.4%) | 83 (31.7%) | 61 (22.9%) | 0.21 |  |
| Acute coronary syndrome | 69 (26.1%) | 79 (30.0%) | 77 (29.2%) | 85 (32.0%) | 0.18 |  |
| History of heart failure | 232 (87.9%) | 236 (89.7%) | 225 (85.2%) | 208 (78.2%) | <0.001 |  |
| Left ventricular ejection fraction, % | 38.0 (13.11) | 36.6 (11.86) | 36.8 (12.73) | 33.5 (11.87) | <0.001 |  |
| Hospitalized for heart failure past year | 56 (21.2%) | 76 (28.9%) | 69 (26.1%) | 68 (25.6%) | 0.39 |  |
| Atrial fibrillation or flutter | 81 (30.7%) | 113 (43.0%) | 139 (52.7%) | 151 (56.8%) | <0.001 |  |
| Systolic blood pressure, mmHg | 123.5 (14.64) | 123.8 (12.41) | 122.8 (11.89) | 121.4 (12.64) | 0.091 |  |
| Pulse, beats/min | 79.8 (11.78) | 77.6 (11.25) | 77.5 (11.26) | 79.5 (12.46) | 0.66 |  |
| BMI (kg/m^2^) | 27.2 (6.22) | 28.3 (6.16) | 28.8 (6.25) | 28.9 (6.17) | <0.001 |  |
| Hemoglobin, g/L | 131.0 (18.05) | 134.9 (19.03) | 138.5 (20.23) | 141.5 (20.94) | <0.001 |  |
| Creatinine, umol/L | 104.1 (31.85) | 107.1 (27.61) | 105.4 (24.80) | 108.1 (29.55) | 0.061 |  |
| Potassium, mmol/L | 4.2 (0.48) | 4.3 (0.41) | 4.3 (0.43) | 4.3 (0.45) | 0.008 |  |
| Sodium, mmol/L | 140.0 (3.77) | 140.2 (4.01) | 140.5 (4.03) | 140.3 (4.76) | 0.076 |  |
| NT-proBNP, ng/L | 3145  (2914, 3395) | 2907  (2708, 3119) | 3275  (3043, 3525) | 3564  (3281, 3870) | 0.004 |  |
| ACE inhibitors/ARBs/ARN inhibitors | 166 (62.9%) | 186 (71.0%) | 170 (64.6%) | 158 (59.6%) | 0.22 |  |
| ß blockers | 95 (36.0%) | 75 (28.6%) | 95 (36.1%) | 109 (41.1%) | 0.082 |  |
| Mineralocorticoid receptor antagonists | 251 (95.1%) | 245 (93.5%) | 251 (95.4%) | 255 (96.2%) | 0.37 |  |
| Furosemide equivalence dose, mg | 68.8 (48.17) | 61.2 (48.93) | 61.0 (44.82) | 60.7 (43.65) | 0.004 |  |
| *reported as n (%) and mean (standard deviation), except for NT-proBNP which is reported as geometric mean (95% CI).  #: Jonckheere’s trend test for continuous variables, Cochron-Armitage trend test for binary variables, CMH general association for categorical variables, and CMH nonzero correlation for ordinal variables. | | | | | |  |

**Supplemental Table 2. Baseline characteristics at time of randomization by quartiles of total bilirubin**

| **Parameter** | **TBil < 10.23**  **umol/L (N=254)** | **TBil  10.23-13.60 umol/L (N=251)** | **TBil  13.67-21.20 umol/L (N=248)** | **TBil  > 21.20**  **umol/L (N=252)** | **Trend**  **P-value#** | |
| --- | --- | --- | --- | --- | --- | --- |
|  |  |  |  |  |  |  |
| Age, years | 61.1 (15.31) | 61.6 (13.89) | 64.4 (12.74) | 63.7 (12.18) | 0.067 | |
| Female sex | 136 (53.5%) | 95 (37.8%) | 93 (37.5%) | 60 (23.8%) | <0.001 | |
| Black race | 87 (34.4%) | 69 (27.5%) | 45 (18.1%) | 17 (6.7%) | <0.001 | |
| Stroke or transient ischemic attack | 27 (10.7%) | 20 (8.0%) | 25 (10.1%) | 21 (8.3%) | 0.54 | |
| Diabetes | 72 (28.5%) | 70 (27.9%) | 72 (29.1%) | 72 (28.7%) | 0.88 | |
| Acute coronary syndrome | 61 (24.0%) | 80 (31.9%) | 79 (31.9%) | 79 (31.3%) | 0.087 | |
| History of heart failure | 209 (82.3%) | 218 (86.9%) | 219 (88.3%) | 211 (83.7%) | 0.56 | |
| Left ventricular ejection fraction, % | 38.7 (13.09) | 37.3 (12.84) | 35.4 (12.12) | 33.7 (12.10) | <0.001 | |
| Hospitalized for heart failure past year | 56 (22.0%) | 47 (18.7%) | 77 (31.0%) | 74 (29.4%) | 0.005 | |
| Atrial fibrillation or flutter | 78 (30.7%) | 94 (37.5%) | 137 (55.2%) | 163 (64.7%) | <0.001 | |
| Systolic blood pressure, mmHg | 124.8 (14.42) | 123.3 (13.30) | 123.1 (12.27) | 119.7 (10.73) | <0.001 | |
| Pulse, beats/min | 79.7 (12.20) | 79.3 (11.39) | 78.4 (11.08) | 77.7 (11.70) | 0.038 | |
| BMI (kg/m^2^) | 27.1 (6.38) | 27.6 (6.17) | 28.7 (6.11) | 29.8 (6.06) | <0.001 | |
| Hemoglobin, g/L | 130.7 (19.09) | 135.5 (18.88) | 137.2 (20.91) | 144.1 (19.23) | <0.001 | |
| Creatinine, umol/L | 105.4 (34.82) | 105.5 (25.54) | 108.4 (26.25) | 108.4 (28.26) | 0.019 | |
| Potassium, mmol/L | 4.2 (0.48) | 4.3 (0.39) | 4.3 (0.45) | 4.3 (0.45) | 0.036 | |
| Sodium, mmol/L | 139.6 (3.60) | 140.7 (3.91) | 140.0 (4.42) | 140.7 (4.44) | 0.006 | |
| NT-proBNP, ng/L | 3155  (2921, 3407) | 2846  (2645, 3062) | 3213  (2961, 3486) | 3588  (3305, 3895) | 0.006 | |
| ACE inhibitors/ARBs/ARN inhibitors | 174 (68.5%) | 145 (58.0%) | 155 (62.8%) | 160 (63.7%) | 0.48 | |
| ß blockers | 80 (31.5%) | 103 (41.2%) | 93 (37.7%) | 92 (36.7%) | 0.37 | |
| Mineralocorticoid receptor antagonists | 234 (92.1%) | 241 (96.4%) | 234 (94.7%) | 242 (96.4%) | 0.069 | |
| Furosemide equivalence dose, mg | 65.9 (55.23) | 57.1 (35.16) | 64.3 (50.98) | 60.3 (40.24) | 0.63 | |
| *reported as n (%) and mean (standard deviation), except for NT-proBNP which is reported as geometric mean (95% CI).  #: Jonckheere’s trend test for continuous variables, Cochron-Armitage trend test for binary variables, CMH general association for categorical variables, and CMH nonzero correlation for ordinal variables. | | | | | |  |

**Supplemental Table 3a. Changes in markers of congestion and changes in ALT from baseline to day 90**

|  | **Change in ALT < -5 U/L (N=326)** | **Change in ALT -5 to +5 U/L (N=373)** | **Change in ALT > +5 U/L (N=197)** | **Spearman Correlation** |  |
| --- | --- | --- | --- | --- | --- |
| **Edema** |  |  |  | Rho=0.09 |  |
| -3 | 2 (0.6%) | 0 | 1 (0.5%) | P=0.008 |  |
| -2 | 12 (3.7%) | 10 (2.7%) | 5 (2.5%) |  |  |
| -1 | 75 (23.0%) | 93 (25.0%) | 41 (20.8%) |  |  |
| 0 | 203 (62.3%) | 231 (62.1%) | 101 (51.3%) |  |  |
| 1 | 32 (9.8%) | 31 (8.3%) | 39 (19.8%) |  |  |
| 2 | 2 (0.6%) | 7 (1.9%) | 9 (4.6%) |  |  |
| 3 | 0 | 0 | 1 (0.5%) |  |  |
| **JVP** |  |  |  | Rho=0.06 |  |
| -2 | 2 (0.7%) | 2 (0.6%) | 1 (0.6%) | P=0.081 |  |
| -1 | 28 (9.6%) | 32 (9.3%) | 14 (8.0%) |  |  |
| 0 | 254 (86.7%) | 299 (86.7%) | 145 (82.9%) |  |  |
| 1 | 8 (2.7%) | 11 (3.2%) | 14 (8.0%) |  |  |
| 2 | 1 (0.3%) | 1 (0.3%) | 1 (0.6%) |  |  |
| **Rales** |  |  |  | Rho=0.15 |  |
| -2 | 8 (2.5%) | 2 (0.5%) | 1 (0.5%) | P<0.001 |  |
| -1 | 32 (9.8%) | 42 (11.4%) | 12 (6.2%) |  |  |
| 0 | 279 (85.6%) | 303 (81.9%) | 152 (78.4%) |  |  |
| 1 | 6 (1.8%) | 23 (6.2%) | 27 (13.9%) |  |  |
| 2 | 1 (0.3%) | 0 | 2 (1.0%) |  |  |
| **NYHA class** |  |  |  | Rho=0.13 |  |
| -3 | 1 (0.3%) | 0 | 0 | P<0.001 |  |
| -2 | 11 (3.4%) | 8 (2.1%) | 2 (1.0%) |  |  |
| -1 | 105 (32.3%) | 101 (27.1%) | 46 (23.4%) |  |  |
| 0 | 183 (56.3%) | 232 (62.2%) | 114 (57.9%) |  |  |
| 1 | 25 (7.7%) | 30 (8.0%) | 27 (13.7%) |  |  |
| 2 | 0 | 2 (0.5%) | 8 (4.1%) |  |  |
| **NT-proBNP#** |  |  |  | Rho=0.10 |  |
| Decrease | 211 (65.5%) | 218 (60.1%) | 104 (53.9%) | P=0.002 |  |
| Stable | 56 (17.4%) | 68 (18.7%) | 31 (16.1%) |  |  |
| Increase | 55 (17.1%) | 77 (21.2%) | 58 (30.1%) |  |  |
| #Patients were grouped according to changes in NT-proBNP as decreased, stable and increased defined as a >=30% decrease, a <30% decrease to <=10% increase, and a >10% increase, respectively. | | | | | |

**Supplemental Table 3b. Changes in markers of congestion and changes in AST from baseline to day 90**

|  | **Change in AST < -5 U/L (N=292)** | **Change in AST -5 to +5 U/L (N=441)** | **Change in AST > +5 U/L (N=164)** | **Spearman Correlation** |
| --- | --- | --- | --- | --- |
| **Edema** |  |  |  | Rho=0.11 |
| -3 | 2 (0.7%) | 0 | 1 (0.6%) | P<0.001 |
| -2 | 12 (4.1%) | 9 (2.0%) | 6 (3.7%) |  |
| -1 | 68 (23.3%) | 113 (25.6%) | 27 (16.6%) |  |
| 0 | 181 (62.0%) | 270 (61.2%) | 85 (52.1%) |  |
| 1 | 28 (9.6%) | 42 (9.5%) | 33 (20.2%) |  |
| 2 | 1 (0.3%) | 7 (1.6%) | 10 (6.1%) |  |
| 3 | 0 | 0 | 1 (0.6%) |  |
| **JVP** |  |  |  | Rho=0.05 |
| -2 | 0 | 4 (1.0%) | 1 (0.7%) | P=0.14 |
| -1 | 26 (10.0%) | 31 (7.6%) | 15 (10.4%) |  |
| 0 | 225 (86.2%) | 365 (89.5%) | 110 (76.4%) |  |
| 1 | 9 (3.4%) | 7 (1.7%) | 17 (11.8%) |  |
| 2 | 1 (0.4%) | 1 (0.2%) | 1 (0.7%) |  |
| **Rales** |  |  |  | Rho=0.13 |
| -2 | 6 (2.1%) | 3 (0.7%) | 2 (1.2%) | P<0.001 |
| -1 | 29 (10.0%) | 44 (10.0%) | 15 (9.3%) |  |
| 0 | 249 (85.6%) | 368 (84.0%) | 116 (71.6%) |  |
| 1 | 6 (2.1%) | 23 (5.3%) | 27 (16.7%) |  |
| 2 | 1 (0.3%) | 0 | 2 (1.2%) |  |
| **NYHA class** |  |  |  | Rho=0.13 |
| -3 | 1 (0.3%) | 0 | 0 | P<0.001 |
| -2 | 10 (3.4%) | 8 (1.8%) | 3 (1.8%) |  |
| -1 | 95 (32.6%) | 119 (27.0%) | 40 (24.4%) |  |
| 0 | 166 (57.0%) | 273 (61.9%) | 87 (53.0%) |  |
| 1 | 19 (6.5%) | 40 (9.1%) | 25 (15.2%) |  |
| 2 | 0 | 1 (0.2%) | 9 (5.5%) |  |
| **NT-proBNP#** |  |  |  | Rho=0.05 |
| Decrease | 178 (62.2%) | 272 (62.8%) | 84 (52.5%) | P=0.10 |
| Stable | 44 (15.4%) | 83 (19.2%) | 27 (16.9%) |  |
| Increase | 64 (22.4%) | 78 (18.0%) | 49 (30.6%) |  |

#Patients were grouped according to changes in NT-proBNP as decreased, stable and increased defined as a >=30% decrease, a <30% decrease to <=10% increase, and a >10% increase, respectively.

**Supplemental Table 3c. Changes in markers of congestion and changes in total bilirubin from baseline to day 90**

|  | **Change in total bilirubin < -3 umol/L (N=314)** | **Change in total bilirubin -3 to +3 umol/L (N=375)** | **Change in total bilirubin > +3 umol/L (N=177)** | **Spearman Correlation** |
| --- | --- | --- | --- | --- |
| **Edema** |  |  |  | Rho=0.11 |
| -3 | 1 (0.3%) | 2 (0.5%) | 0 | P=0.001 |
| -2 | 7 (2.2%) | 12 (3.2%) | 5 (2.8%) |  |
| -1 | 86 (27.4%) | 87 (23.3%) | 31 (17.5%) |  |
| 0 | 186 (59.2%) | 231 (61.8%) | 99 (55.9%) |  |
| 1 | 29 (9.2%) | 38 (10.2%) | 33 (18.6%) |  |
| 2 | 5 (1.6%) | 4 (1.1%) | 8 (4.5%) |  |
| 3 | 0 | 0 | 1 (0.6%) |  |
| **JVP** |  |  |  | Rho=0.02 |
| -2 | 0 | 3 (0.9%) | 2 (1.3%) | P=0.66 |
| -1 | 25 (9.0%) | 33 (9.6%) | 14 (8.8%) |  |
| 0 | 243 (87.1%) | 296 (86.0%) | 130 (81.8%) |  |
| 1 | 11 (3.9%) | 9 (2.6%) | 13 (8.2%) |  |
| 2 | 0 | 3 (0.9%) | 0 |  |
| **Rales** |  |  |  | Rho=0.06 |
| -2 | 1 (0.3%) | 6 (1.6%) | 1 (0.6%) | P=0.092 |
| -1 | 27 (8.7%) | 37 (9.9%) | 14 (8.0%) |  |
| 0 | 270 (86.8%) | 308 (82.6%) | 140 (79.5%) |  |
| 1 | 13 (4.2%) | 21 (5.6%) | 19 (10.8%) |  |
| 2 | 0 | 1 (0.3%) | 2 (1.1%) |  |
| **NYHA class** |  |  |  | Rho=0.06 |
| -3 | 1 (0.3%) | 0 | 0 | P=0.078 |
| -2 | 5 (1.6%) | 13 (3.5%) | 3 (1.7%) |  |
| -1 | 94 (30.0%) | 98 (26.1%) | 50 (28.2%) |  |
| 0 | 188 (60.1%) | 230 (61.3%) | 92 (52.0%) |  |
| 1 | 23 (7.3%) | 33 (8.8%) | 25 (14.1%) |  |
| 2 | 2 (0.6%) | 1 (0.3%) | 7 (4.0%) |  |
| **NT-proBNP#** |  |  |  | Rho=0.12 |
| Decrease | 205 (66.3%) | 229 (62.4%) | 87 (50.0%) | P<0.001 |
| Stable | 45 (14.6%) | 66 (18.0%) | 29 (16.7%) |  |
| Increase | 59 (19.1%) | 72 (19.6%) | 58 (33.3%) |  |

#Patients were grouped according to changes in NT-proBNP as decreased, stable and increased defined as a >=30% decrease, a <30% decrease to <=10% increase, and a >10% increase, respectively.

**Supplemental Figure 1. Relative hazard of death or HF readmission through Day 180 by baseline log-transformed liver function tests modelled as a log-linear term**

**
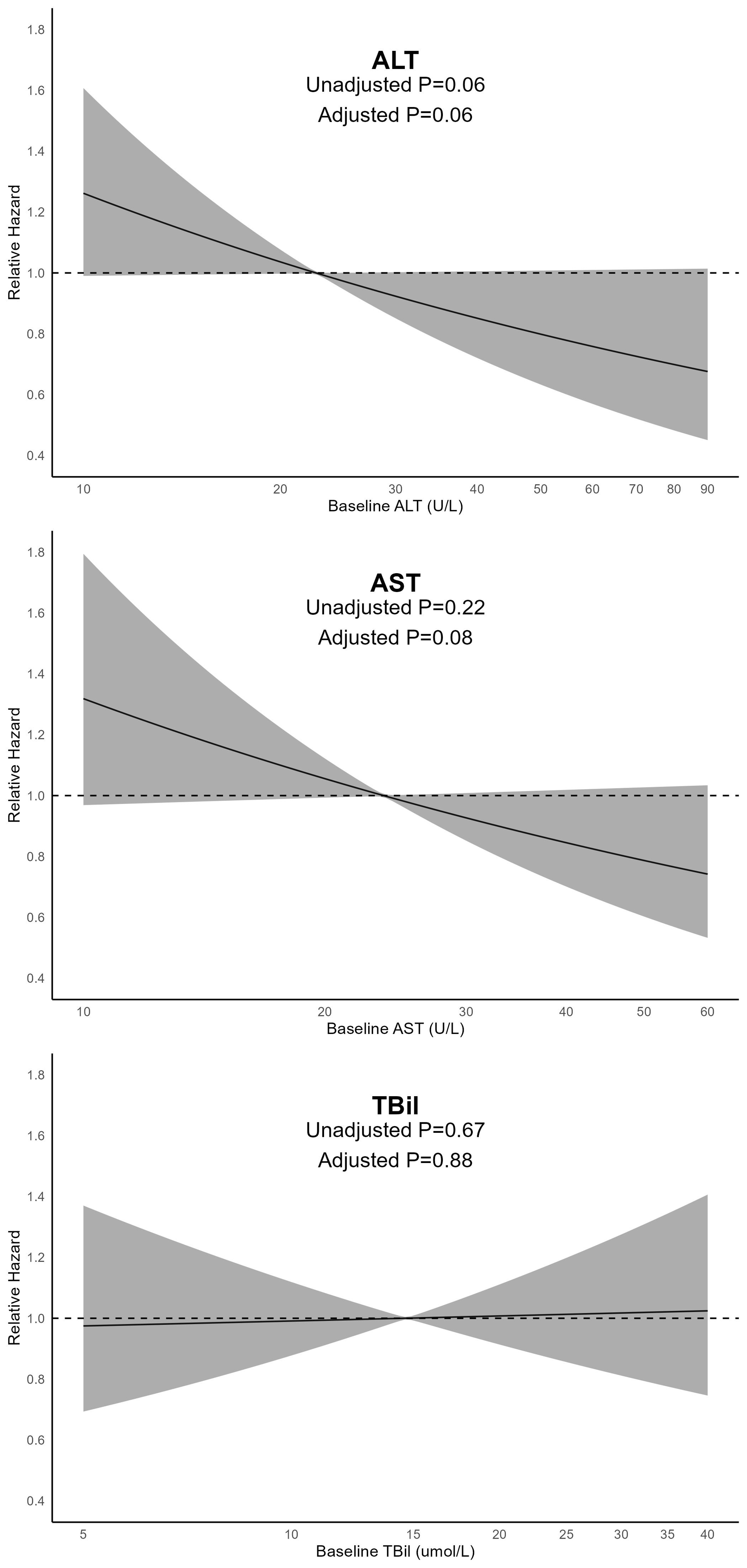
**

**Supplemental Figure 2. Association of baseline LFT values with change in EQ-5D VAS from baseline to day 90 adjusted for baseline EQ-5D VAS**

| 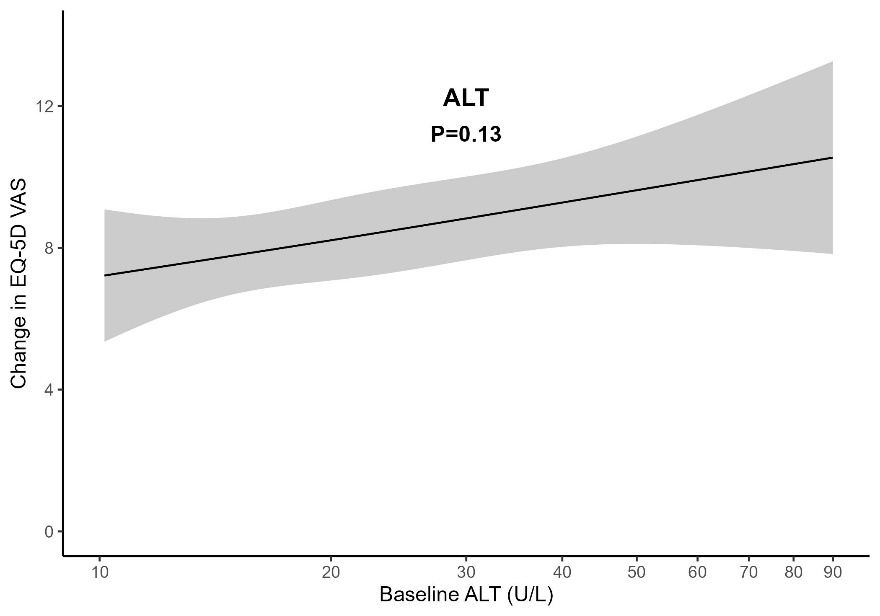 |
| --- |
| 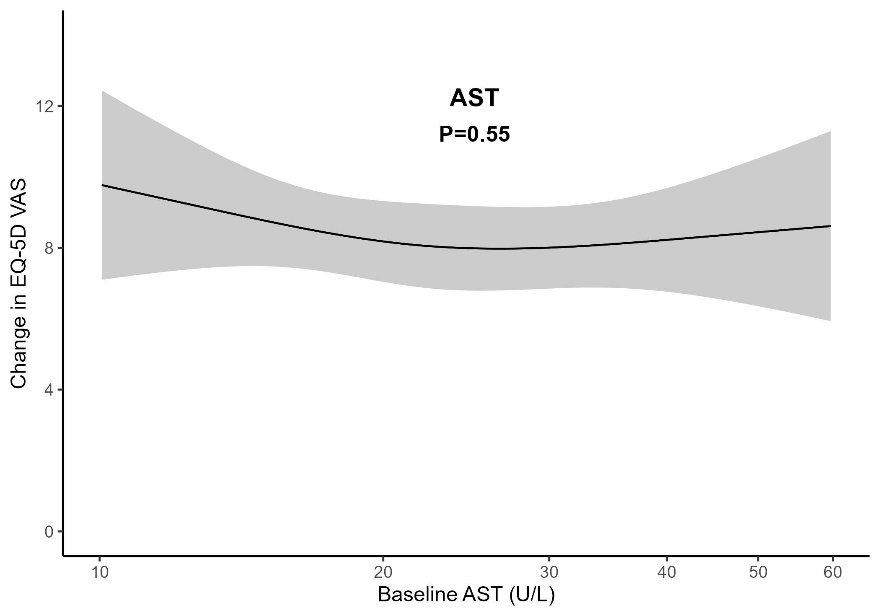 |
| 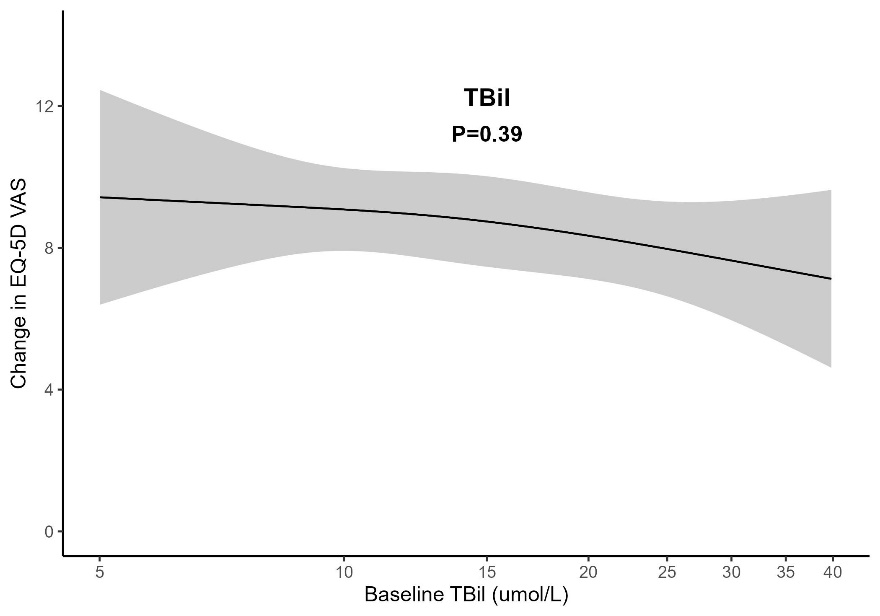 |

**Supplemental Figure 3. Treatment effect of high intensity care vs. usual care on change in EQ-5D VAS from baseline to day 90 according to baseline LFT values adjusted for baseline EQ-5D VAS**

| **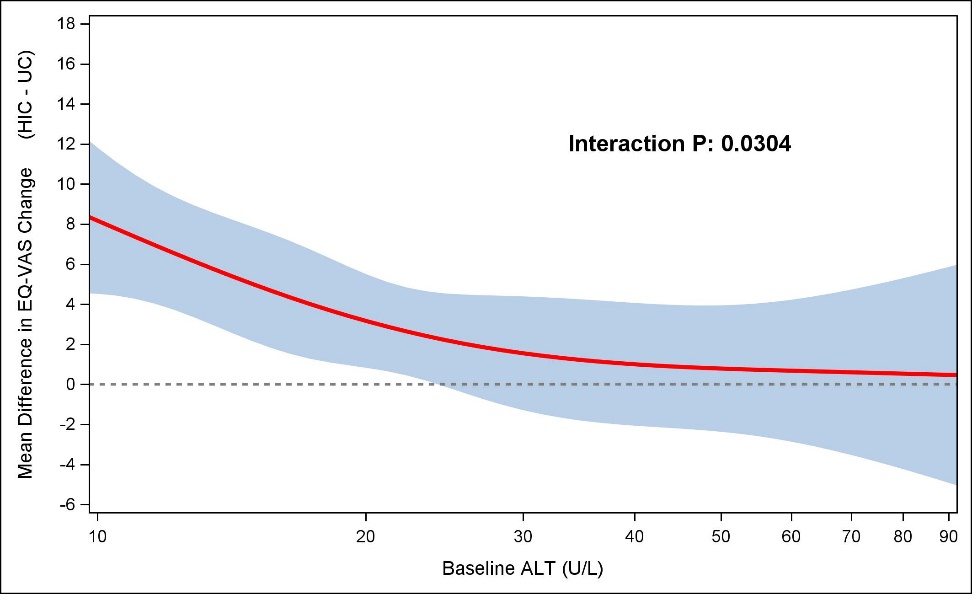** |
| --- |
| **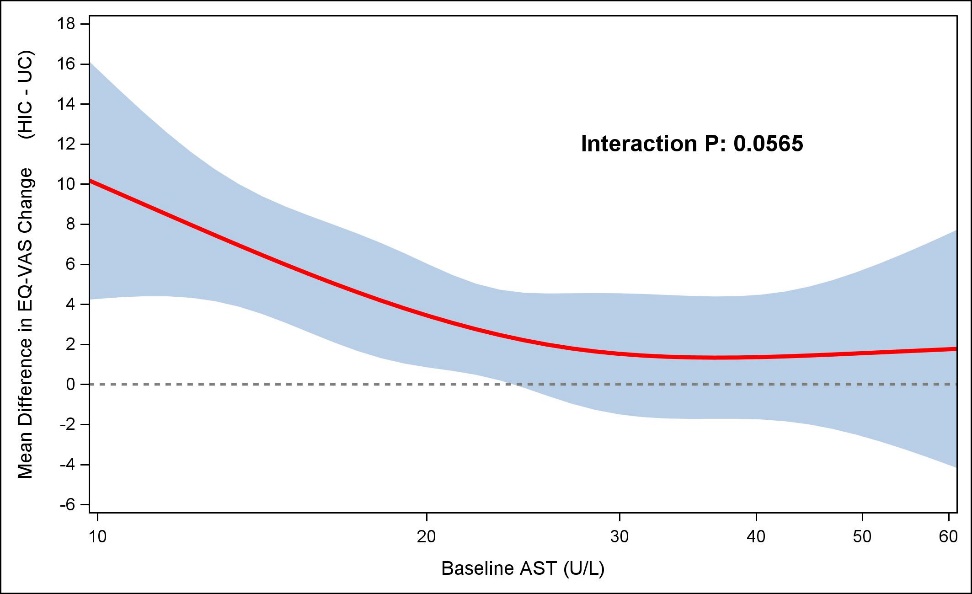** |
| **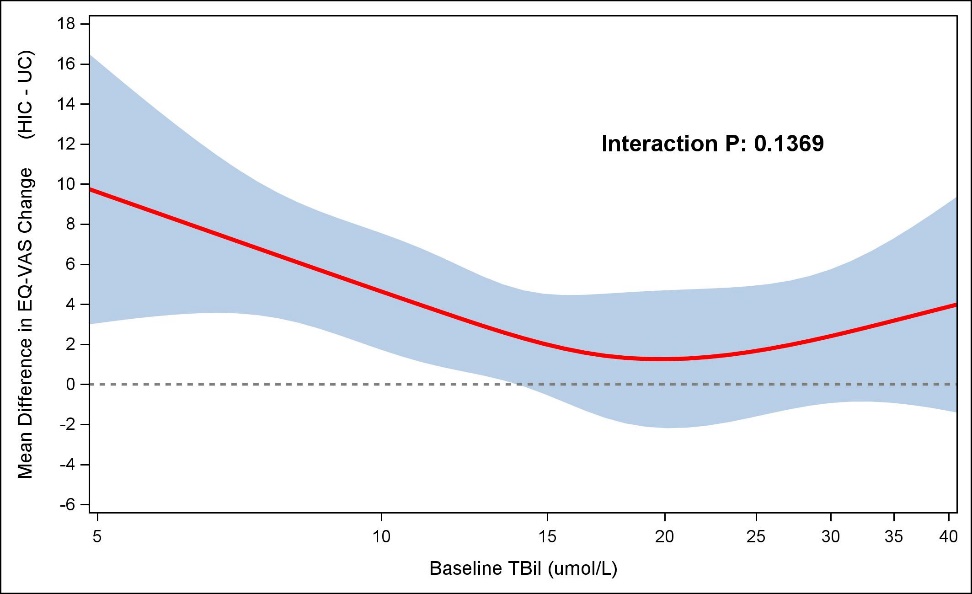** |
